# Patient Perception of Medical Learners and Medical Education during Clinical Consultation at a Family Medicine Residency

**Published:** 2018-11-29

**Authors:** Kyle Goerl, Samuel Ofei-Dodoo

**Affiliations:** University of Kansas School of Medicine-Wichita, Department of Family and Community Medicine

**Keywords:** patient preference, medical education, family practice

## Abstract

**Introduction:**

Experience in treating patients under supervision of faculty is an important factor in medical education at all levels. However, unpleasant patient experiences with a medical learner during clinical consultation can damage the relationship between the medical learner, physician supervisor, and patient. A goal of this study was to examine patient experiences and preferences regarding medical learners during clinical consultation at a family medicine residency clinic. Another goal was to determine factors relating to patients’ experiences and preferences regarding medical learners.

**Methods:**

This cross-sectional study relied on patients completing a survey designed from extant questionnaires to measure patients’ experiences and preferences relating to interactions with medical learners at a family medicine clinic. Data were collected from 216 patients between December 2016 and August 2017. We correlated patients’ feelings, overall experiences with medical learners and the importance of medical education.

**Results:**

There was a 93% participation rate. The patients rated their overall experiences with medical learners as 3.8 on a 5-point scale, suggesting positive experiences. Eighty-eight percent prefer not more than three medical learners to be involved in their care during clinical consultation. Patients’ overall experiences with medical learners participating in medical care correlated with their preferences regarding medical learners’ involvement in their treatment (r[209] = .524; p = 0.01). Patients’ perception of medical learners participating in medical care correlated with the importance of medical education (r[209] = .878; p = 0.01).

**Conclusions:**

The results showed that most patients have positive experiences with medical learners and are generally in favor of medical education.

## INTRODUCTION

As medical education curricula around the U.S. develops, one consistent movement is the drive to provide earlier patient encounters for medical learners. For the purposes of this study, medical learners are individuals in training to become physicians, including residents and medical students. Medical learner and patient interactions come in real and simulated scenarios, both with their own merits. Real patient experiences have been shown to be more authentic and instructive, whereas simulated patients prepare learners for real life encounters, practice sensitive exams, and obtain feedback in a safe environment.^1^ This interaction provides context to what medical learners learned in the classroom and helps them learn clinical, communication, and professional skills. Patient exposure in the preclinical years, even in a classroom setting, has been shown to enhance empathy, improve knowledge retention, and bring joy to the participating learners.^2^

What requires further investigation is how these experiences affect patients. There is a relative paucity of research in this area. The research that exists showed the relationship between patients and learners to be positive.^3^–^6^ Recent data in the primary care literature suggested that patients were satisfied with encounters where medical learners are involved, but patients were less inclined to share sensitive or personal issues with their personal physicians when medical learners are present.^7^

The aforementioned discussion demonstrated that the educational experience for the learner is enhanced by interactions with real patients.^8^ However, this relationship could be damaged when patients’ needs conflict with medical educational requirements,^9^ especially when patients have unpleasant experiences with medical learners during health care consultation. Therefore, the current study sought to: (1) explore patients’ experiences with medical learners and learn about patients’ overall views toward the presence of medical learners during consultation at a medical educational clinic; (2) identify patients’ preference regarding medical learners’ involvement in their treatment; and (3) find factors that relate to patients’ experiences and preferences regarding medical learners’ involvement in patients’ treatment.

## METHODS

### Study design

This cross-sectional study involved adult patients completing a survey after their outpatient clinic visits at the University of Kansas School of Medicine-Wichita Family Medicine Residency Program at Via Christi Hospitals. The clinic is one of three family medicine residency programs that serves the healthcare needs of people in the Wichita area and rural Kansas.^10^ The Via Christi Health and the University of Kansas School of Medicine-Wichita Institutional Review Boards approved the study. A sample size of 100 was calculated as necessary for adequate power (> .85) to detect significant correlations of 0.5, p < 0.01 between variables.^11^

### Procedure

Adult patients checking out after their clinic visits, who had experiences with medical learners (residents and/or medical students) during their clinic consultations, were asked to participate. Extant questionnaires^12^–^14^ were tailored to the study purposes. Data were collected from 216 patients from December 2016 to August 2017. Patient identification was not collected.

### Data analysis

Standard descriptive summary statistics were used to examine patients’ perception of medical learners. We used correlations to determine association between patients’ experiences with medical learners and the importance of medical education. A statistical critical value of 0.05 was specified for all tests.

## RESULTS

Two hundred twenty-two patients met the inclusion criteria; 216 agreed to participate in the study for a participation rate of 97.3%. The average age of participants was 38.6 ± 17.6 years (Table 1). There were more women (59%) than men in the sample; 76% were Caucasians; 49% had never been married; and 26% had bachelor’s degrees. The study findings are summarized in Tables 2 – 5. Generally, the patients had positive feelings about medical learners’ involvement in their care, but 51% had no opinion as to whether medical learners involvement in care improves the physician supervisor’s competence, while 49% of the patients were neutral on whether medical learners involvement in the care improved the quality of care they receive (Table 2). As shown in Table 3, some patients expressed concerns about the group size of the medical learners. In particular, 88% would not allow more than three medical learners to be present while being examined during clinical consultation (Table 3). Eighty-six percent indicated that they would allow medical learners to be present while discussing medical concerns with their attending doctor.

Similarly, most patients expressed that their encounters with medical learners are important for future training of medical doctors. While 45.5% of the patients reported that they do not mind the presence of medical learners during clinical consultation, 85.3% indicated that involvement of medical learners in patient care is very important/important for medical education (Table 4). As shown in Table 5, the patients’ overall experiences with medical learners participating in medical care significantly correlated with the (1) patients’ preferences regarding medical learners’ involvement in their treatment and (2) importance of training future doctors. In addition, patients’ perception of medical learners participating in medical care correlated with the importance of medical education (r[209] = .878; p = 0.01).

## DISCUSSION

The study provided information regarding patients’ experiences, preferences, and attitudes about medical learners during clinical consultation at a family medicine residency program. The findings demonstrated that most patients not only have positive perceptions about medical learners, they think that having the medical learners participate in their care is important in medical education. These data appeared to confirm previous findings that have showed a majority of patients do not object to medical learners participating in their care.^12^,^15^ The study findings should be reassuring to community physicians who take medical learners in their practices. Having learners present during clinical consultation is generally a positive experience for all parties involved, and it is unlikely to affect the relationship between the attending physician and their patients.

A major finding of the study demonstrated the importance of group size on patient preferences. The majority of patients preferred no more than three medical learners to participate in their care. This finding is consistent with another study,^14^ and it is important information, as it allows medical educators to plan for appropriate group sizes for clerkships in outpatient family medicine practices. More than half of the patients have no opinion while 27% thought that involvement of medical learners in patient care improves the physician supervisor’s competence, suggesting that the patients did not think the presence of the medical learners would affect the physician supervisor’s competency. This revelation is similar to the finding of a previous study where many patients had no opinion or thought medical learners’ involvement in patient care improves physician supervisor’s competence.^12^ Our study also highlighted the presence of medical learners and its effects on quality of care patients receive. Almost half of the patients did not think medical learners’ involvement affected the quality of care they receive. Consistent with previous studies,^16^,^17^ our data supported the assertion that medical learners’ involvement in patient care did not affect the quality of care patients receive at residency clinics adversely.

The study had limitations. First, it was conducted in an outpatient family medicine practice, potentially limiting generalizability to other contexts, such as inpatient interactions. It would be worth determining if these findings were consistent across all specialties, as well as inpatient versus outpatient scenarios. This study also was limited in its diversity. It was conducted in a practice where a majority of the participants were Caucasian. Based on cultural beliefs, patients from other ethnic groups might have different opinions regarding medical learners’ participation in their care. Further research is needed to determine if the findings are consistent across all races and cultures. Patients’ self-reported clinical experiences also limit the findings of the study.

In conclusion, this study has drawn attention to patients’ experiences with medical learners in the clinical setting. The overall positive patient perception of medical learners should be comforting to physicians who teach, recognizing that having medical learners participate in patient care has little negative impact on their patients’ perception of care. The positive correlation between patients’ overall experiences with medical learners and views regarding medical education suggested that patients will be in favor of medical education when they have better experiences with medical learners.

## Figures and Tables

**Table 1:** Demographic profile of participants.

Demographic of Participants	Measure
Sex	
Male	40.8 (87)
Female	59.2 (126)
Missing	3
Age (years)	
Range	18 to 84
Mean (SD)	38.6 (17.6)
Ethnicity/Race	
African American	12.3 (26)
Caucasian	75.9 (161)
Hispanic/Latino	5.2 (11)
Asian	2.4 (5)
Bi-racial	2.4 (5)
Other	1.9 (4)
Missing	3
Marital Status	
Single (never married)	48.8 (103)
Married	45.5 (96)
Separated/divorced	2.8 (6)
Widow/widower	2.8 (6)
Missing	5
Educational Level	
No high school	3.8 (8)
Did not complete high school	21.8 (46)
Graduated from high school	6.2 (13)
Some college	18.0 (38)
Technical	2.4 (5)
Associate’s degree	8.1 (17)
Bachelor’s degree	25.6 (54)
Master’s degree	11.4 (24)
Doctorate degree	2.8 (6)
Missing	5

Data are % (n) unless otherwise indicated.

**Table 2:** Patients’ experiences with medical learners during family medicine clinical rotations.

	Strongly Disagree	Disagree	Neutral	Agree	Strongly Agree	Score
	(Scoring 1)	(Scoring 2)	(Scoring 3)	(Scoring 4)	(Scoring 5)	Mean
**Patient Experiences Items (α = 0.69) (N = 216)**	%	%	%	%	%	(SD)
How much do you agree with the following statements:						
Seeing the medical learner is enjoyable.	1.4	0.9	21.8	37.0	38.9	4.1 (0.9)
Having medical learner participate takes too much time (reverse scoring).	3.2	3.7	19.4	35.2	38.4	4.0 (1.1)
Having medical learner involved interferes with the relationship I have with my doctor (reverse scoring).	1.9	2.8	17.1	36.1	42.1	4.1 (0.9)
Having medical learner participate decreases my time with my doctor (reverse scoring).	1.4	6.0	19.0	38.9	34.7	4.0 (1.0)
Having medical learner involved improves my doctor’s competence.	6.0	15.7	50.9	17.1	10.2	3.1 (1.0)
Having medical learner involved improves the quality of care I receive.	4.6	6.9	46.8	24.1	17.6	3.4 (1.0)
**Overall Patient Experiences**						**22.8 (3.6)**

**Table 3:** Patients’ preferred number of medical learners to be present and/or examine them during clinical consultation.

	Present During Consultation		Examine During Consultation	
# of Medical Learners	Number	Percentage	Number	Percentage
0	13	6.2	16	7.6
1 – 3	181	86.2	183	87.6
4 – 8	9	4.3	4	1.9
More than 8	7	3.3	6	2.9
**Total**	**210**	**100.0**	**209**	**100.0**

**Table 4:** Respondents’ perception about medical learners and medical education.

	Possible Category and Scoring						
	Very Comfortable^a^ or Very Important^b^	Comfortable^a^ or Important^b^	Do Not Mind^a^ or Not Sure^b^	Uncomfortable^a^ or Unimportant^b^	Very Uncomfortable^a^ or Very Unimportant^b^		Score
	(Scoring 1)	(Scoring 2)	(Scoring 3)	(Scoring 4)	(Scoring 5)	Missing	Mean
**Questions**	%	%	%	%	%	*n*	SD
How do you feel about medical learners being present while you are talking to the doctor about your problem?^a^	25.8	23.5	45.5	4.2	0.9	3	2.3 (0.9)
How important for the training of future doctors is it that medical learners are present while patients are seeing their doctors?^b^	62.4	22.4	13.8	1.4	-	6	1.5 (0.8)
How important for the future training of doctors do you think it is that medical learners examine patients?^b^	60.7	25.1	13.3	0.5	0.5	5	1.6 (0.8)

Possible score range for all scales: 0 – 100.

a response categories for items denoted with a.

b response categories for items denoted with b.

**Table 5:** Correlations of respondents’ experiences with medical learners and the importance of medical education.

Measure		1	2	3	4
1. How do you feel about medical learners being present while you are talking to the doctor about your problem?	Pearson Correlation	-			
	Sig. (2-tailed)				
	N				
2. How important for the training of future doctors is it that medical learners are present while patients are seeing their doctors?	Pearson Correlation	0.405**	_		
	Sig. (2-tailed)	0.000			
	N	210			
3. How important for the future training of doctors do you think it is that medical learners examine patients?	Pearson Correlation	.445**	.878**	-	
	Sig. (2-tailed)	0.000	0.000		
	N	211	210		
4. Overall patients’ experiences with medical learners during clinical consultation as indicated in Table 2.	Pearson Correlation	.407**	.492**	.524**	-
	Sig. (2-tailed)	0.000	0.000	0.000	
	N	213	210	211	
	Mean	2.31	1.54	1.55	22.79
	Standard Deviation	0.94	0.78	0.78	3.62
	Range	1 – 5	1 – 5	1 – 5	6 – 30

** Correlation is significant at the 0.01 level (2-tailed).
